# Impact of Submicroscopic *Plasmodium falciparum* Parasitaemia on Maternal Anaemia and Low Birth Weight in Blue Nile State, Sudan

**DOI:** 10.1155/2019/3162378

**Published:** 2019-08-07

**Authors:** Samia A. Omer, Ali N. Noureldein, Hadeel Eisa, Mutasim Abdelrahim, Hagir E. Idress, Abdelrahim M. Abdelrazig, Ishag Adam

**Affiliations:** ^1^Department of Immunology and Molecular Biology, Tropical Medicine Research Institute, National Centre for Research, Khartoum, Sudan; ^2^Faculty of Medicine, University of Khartoum, Khartoum, Sudan; ^3^Ed-Damazin Hospital, Blue Nile State Ministry of Health, Ed-Damazin, Sudan; ^4^School of Biomedical Sciences, Middlesex University, London, UK; ^5^Department of Microbiology, Faculty of Medicine, Blue Nile University, Ad-Damazīn, Sudan

## Abstract

The aim of the present study was to investigate the prevalence of submicroscopic infections and to assess its impact on maternal anaemia and low birth weight. A cross-sectional study was carried out with 1149 consented pregnant women who delivered at 3 main hospitals in the Blue Nile State, between January 2012 and December 2015. From a matched maternal peripheral, placental maternal side, and cord blood sample, blood films and dried spots were prepared for microscopic examination and nested polymerase chain reaction (n-PCR), respectively. 107 out of 447 negative blood films were found to have submicroscopic infection detected using n-PCR in any of the three compartments. Placental samples had a significantly higher prevalence (142) of submicroscopic infections compared with the peripheral (6.5%) and cord (8.1%) samples. The mean (SD) of the maternal haemoglobin (Hb) was significantly lower in cases with submicroscopic parasitaemia (10.9 (0.8) vs. 12.1 (0.7) g/dl, *P* < 0.001) compared with those who had no submicroscopic parasitaemia. Submicroscopic malaria infection was associated with anaemia (OR 19.7, (95% CI, 10.3–37.8)). Thirty-eight babies born to women with submicroscopic infections were low birth weight (LBW) and was significantly lower in submicroscopic parasitaemia (2.663 (0.235) vs. 2.926 (0.341) kg, *P* < 0.001). Submicroscopic malaria infection was associated with LBW (OR = 2.7, (95% CI, 1.2–5.6)). There is a high incidence of submicroscopic infections in any of the three compartments regardless of age or parity. Submicroscopic infection is a risk of maternal anaemia and low birth weight in women in this area of high seasonal malaria transmission.

## 1. Introduction

Each year, 50 million women living in malaria-endemic areas become pregnant; in 2015, an estimated 28 million pregnant women were at risk of malaria in the sub-Saharan Africa [1]. Pregnant women are particularly vulnerable to malaria infection, which increases the risk of poor maternal and newborn outcomes including severe maternal anaemia, maternal death, spontaneous abortion, low birth weight, and newborn and infants deaths [2–4].

The placenta provides an ideal environment for the sequestration of parasite-infected red blood cells that adhere to the adhesion molecule Chondroitin sulfate A (CSA) receptors in the placental syncytiotrophoblast with the parasite ligand VAR2CSA [5, 6]. Molecular detection methods of *Plasmodium*, particularly the polymerase chain reaction (PCR) method, have been shown to be significantly more sensitive in detecting low-level parasitaemia than the standard diagnostic method of thick blood film microscopy in both peripheral blood and placental blood [7–9].

Different rates and determinants of submicroscopic parasitaemias during pregnancy have been reported in the different African regions [10–13], and outside Africa [8, 14].

It has been documented that submicroscopic infections are associated with decreased maternal haemoglobin (Hb) and low birth weight (LBW) in areas with different levels of malaria transmission [11, 15–17].

It has been shown that peripheral, placental, and submicroscopic malaria infections during pregnancy are a big health problem in different areas of Sudan [18–20]. Approximately, more than quarter of the pregnant Sudanese women who had been found to be smear-negative for malaria had PCR-based evidence of submicroscopic *P*. *falciparum* infection [12, 21, 22]. Low-level parasitaemia during pregnancy is often not recognized or is underestimated using microscopy as many of the *P*. *falciparum* infections are asymptomatic and remain undetected and untreated in routine antenatal care (ANC). The purpose of this work was to determine the rate of the submicroscopic malaria infections and to investigate its association with maternal anaemia and LBW in Blue Nile state, Sudan, which is characterized by intense malaria transmission.

## 2. Materials and Methods

As part of a study on placental malaria conducted in Blue Nile state, Sudan, samples were collected according to Omer et al. [20]. Briefly, a cross-sectional study was carried out in the Blue Nile state (9°30 and 35°3 east) which is an area of 45,844 km^2^ and has an estimated population of 832,000. The study was conducted at the same time in three main maternity units of the hospitals: Ed-Damazin Hospital, El-Roseures Hospital, and the Surgical Complex from January 2012 to December 2015. A total of 1149 women fulfilled the inclusion criteria consented to participate the study. Data including age, gravidity, education, and residence were collected in a predesigned questionnaire. Hb concentrations were estimated, and anaemia was defined as Hb < 11 g/dL. Newborn babies were weighed immediately following birth using an electronic scale to the nearest 10 grams, and the sex of the neonates was recorded. Newborns were classified as normal birth weight (≥2.5 kg) or LBW (<2.5 kg).

### 2.1. Blood Samples Collection and Analysis

From a matched maternal peripheral, placental maternal side, and cord blood sample, blood films and dried spots were prepared for microscopic examination and molecular analysis, respectively. A finger prick was used to test for malaria microscopy and to measure Hb level. About 3–5 drops of blood were spotted on a Whatman® 3 mm filter paper (Whatman International Ltd, Maidstone, UK) for nested PCR (n-PCR) analysis. Each filter paper sample was dried at room temperature and stored at 4°C in a separate plastic bag to avoid cross contamination for subsequent DNA extraction.

Thin and thick blood films were stained with 10% Giemsa for 10 minutes and examined by experienced microscopists under 100 oil immersion lens. A slide was considered negative after examination of at least 100 microscopic fields. Microscopic examination of thick films, using high power magnification, was performed to detect the presence of parasites, while the thin films were used to determine the species when thick films were positive.

### 2.2. Molecular Detection

Parasite DNA was extracted from the dried spots of the three compartments using saponin-Chelex method as described by Plowe et al. [23]. A circular punch of about 6 mm of each filter paper corresponding to approximately 25 *μ*l blood was placed in 1 ml of phosphate buffered saline (PBS) containing 0.5% saponin and incubated overnight at 4°C. The resulting brown solution was replaced with 1 ml PBS and incubated for an additional 15–30 minutes at 4°C. After this incubation, 200 *μ*l of 20% Chelex®100 (Sigma, UK) was placed in clean tubes and heated at 100°C in a water bath. The disks were removed from the PBS and placed in the preheated 20% Chelex®100, vortexes at high speed for 30 seconds and placed in a water bath at 100°C for 10 minutes with gentle agitation. Samples were then centrifuged at 10,000 × g for 5 minutes, and the supernatant was removed and centrifuged as before. The supernatant was then collected into a clean tube and used for PCR immediately or stored at 4°C (or at 20°C) until use. A volume of 5 *μ*l was used as a template for amplification of 18s ribosomal RNA (rRNA) gene of *P*. *falciparum*. As *P*. *vivax* is not common and other *P*. spp. is not reported, all samples were checked for *P*. *falciparum* infection.

PCR was carried out using outer and nested primers to enhance detection of low-density *P*. *falciparum* infections below the threshold of microscopy in an n-PCR using genus-specific primers for primary PCR reaction followed by species detection using the amplified product [24]. PCR assays were performed on coded samples blinded to the results of microscopy. Samples were considered positive when species-specific primers generated a positive indication. Samples were processed once as long as controls were confirmed to operate in optimal conditions; otherwise, the assay was repeated until the successful performance of controls. PCRs were considered positive if a specific band of the expected base pair (bp) size was observed.

### 2.3. Statistics

Data analysis was performed using SPSS version 20.0. Mean (SD) and proportions were compared between the two groups using *t*-test *X*^2^, respectively. Binary regression analyses were performed where the submicroscopic parasitaemia, maternal anaemia, and LBW were entered separately in each model as dependent variable and age, parity, residence, antenatal care, using bednets, and newborn sex as independent variables. The submicroscopic parasitaemia itself was entered as independent variable in the models when anaemia and LBW were the dependent variables. ORs and 95% CI were calculated, and *P* < 0.05 was considered significant.

## 3. Results

### 3.1. General Characteristics

Four hundred forty-seven cases had blood films that were negative in all 3 compartments and had complete data; therefore, they were considered for submicroscopic parasitaemia analyses. The mean (SD) age and parity of the 447 enrolled women were 26.9 (4.5) years and 3.4 (2.0), respectively. Seventy-nine (17.7%) of the 447 enrolled women were primiparous. The majority of the women were rural residents (81.8%), who had <secondary educational levels (70.0%) and had no antenatal care (74.5%). Only 80 (17.9%) of these women had used bednets in the index pregnancy. Over half (58.8%) of the women had a history of malaria (unconfirmed) in the index pregnancy.

The mean (SD) of Hb and birth weight was 11.4 (1.8) g/dl and 2.840 (0.33) kg, respectively. Two hundred eighty-five (63.8%), 103 (23.0%), and 59 (13.2%) women were recruited from the Surgical Complex, El Roseires Hospital, and Ed-Damazin Hospital, respectively.

Out of these 447, 147 (32.9%) had submicroscopic parasitaemia (detected in any of the three compartments). The prevalence of submicroscopic parasitaemia (blood films were negative) was significantly higher in the placenta: 29 (6.5%), 142 (31.8%), and 36 (8.1%) (*P* < 0.001) in the peripheral, placental, and cord samples, respectively.

Only 9 (2.0%) cases of submicroscopic parasitaemia were detected in the three compartments of the peripheral, placenta, and cord samples. Submicroscopic parasitaemia was detected in 25 (5.6%) samples in both peripheral and placental samples.

Residence, not attended ANC, and not using bednets were associated with submicroscopic parasitaemia in univariate analyses. Binary regression analysis showed no association between submicroscopic parasitaemia and age, parity, residence, antenatal care, and use of bednets; however, women with education less than secondary level were at higher risk for submicroscopic parasitaemia (OR = 4.1, 95% CI = 2.3–7.4; *P* < 0.001) (Table 1).

### 3.2. Maternal Anaemia

The mean (SD) of the maternal Hb was significantly lower in cases with submicroscopic parasitaemia (10.9 (0.8) vs. 12.1 (0.7) g/dl, *P* < 0.001). Compared with subjects who had no submicroscopic parasitaemia, significantly higher number of cases with submicroscopic parasitaemia had anaemia [75/147 (51.1%) vs. 16/300 (5.3%), *P* < 0.001].

In binary regression, age, parity, residence, education, and not using bednets were not associated with maternal anaemia; however, submicroscopic parasitaemia was the only factor associated with maternal anaemia (OR = 19.7, 95% CI = 10.3–37.8; *P* < 0.001) (Table 2).

### 3.3. Low Birth Weight

Of 447 cases, 38 (8.5%) were LBW. The birth weight was significantly lower in cases with submicroscopic parasitaemia (2.663 (0.235) vs. 2.926 (0.341) kg, *P* < 0.001).

Compared with subjects who had no submicroscopic parasitaemia, significantly higher number of cases with submicroscopic parasitaemia had LBW (22/147 (15.0%) vs. 16/300 (5.3%), *P*=0.001).

In binary regression, age, parity, residence, education, and not using bednets were not associated with LBW; however, no ANC and submicroscopic parasitaemia were the factors associated with LBW (OR = 2.7, 95% CI = 1.2–5.6; *P*=0.008) (Table 3).

## 4. Discussion

The present study is the first to assess the rates of the submicroscopic *P*. *falciparum* infection and its associations with the adverse pregnancy outcomes in Sudanese women from Blue Nile state, a high seasonal malaria transmission. A marked underestimation of malarial infection in pregnant women when diagnosed by standard microscopy in maternal peripheral, placental, or cord blood compared with PCR was found (an overall 32.9% submicroscopic parasitaemia).

In comparison with the peripheral (6.5%) and cord (8.1%) samples, placental samples had a significantly higher prevalence (31.8%) of submicroscopic *P*. *falciparum* infections (*P* < 0.001). This finding is in line with the previous studies in Sudan on the frequency of submicroscopic infection in the three compartments [12, 25]. Other reports described submicroscopic parasitaemia in different geographical areas and in a systemic review carried out in Africa; the weighted mean of submicroscopic infection was 36% [16]. In Gabon, 23% of the subjects have submicroscopic malaria infection in both the placental blood and peripheral blood [15]. However, only 9.7% of the women who were originally negative by microscopy were reported to have submicroscopic malaria infection in Ghana may be as a result of the implementation of malaria control measures [26]. In a study performed in Colombia, the frequency of malaria infection in the three compartments varied significantly between microscopy and PCR with 65% of the pregnant women had at least one positive compartment [14]. In India, nearly two-thirds of both peripheral and placental infections were identified as submicroscopic parasitaemia [9]. This suggests low-density infections and free circulating DNA molecules could be detected using highly sensitive molecular techniques rather than microscopy. However, Okell and colleagues [27] found a significant association between the transmission intensity and the proportion of infections that are submicroscopic and higher percentage of total infections to be detected by microscopy in areas of high, compared with low, transmission, although there is some evidence that low-density parasites might serve as a reservoir for continued transmission of malaria and individuals with submicroscopic malaria can infect mosquitoes [28]. Therefore, the extent of the submicroscopic reservoir needs to be taken into account for effective surveillance and control programmes and treatment.

The present study showed no association between the submicroscopic parasitaemia and age and parity. This is consistent with previous studies performed in an area of unstable malaria transmission in Eastern Sudan [21, 25]. Likewise, age and parity were not associated with submicroscopic malaria in an area with intense malaria transmission in Gabon [29], and in Cameron and Malawi [11, 30]. However, the prevalence of submicroscopic infections was associated with primigravidity in Malawi [11] and Uganda [13] but not in Ghana [26]. Our study area is of high seasonal transmission; this suggests an enhancement of immune protection limiting *P*. *falciparum* to submicroscopic levels.

In the current study, cases with submicroscopic parasitaemia were 19.7 at higher risk to have anaemia. Previous studies from Mozambique [7], Cameroon [30], and Ghana [26, 31] reported a significant association between submicroscopic malaria infection and low maternal Hb levels/anaemia. However, findings from Mozambique [32] and Sudan [21] reported a nonsignificant association between anaemia and submicroscopic parasitaemia.

The results presented here found submicroscopic parasitaemia is a risk factor (OR = 2.7, 95% CI = 1.2–5.6) for LBW. This is consistent with prior findings in unstable malaria transmission in Sudan where birth weights were significantly lower in women who had submicroscopic parasitaemia [12]. Adegnika et al. [15] reported women with submicroscopic *P*. *falciparum* infections in Gabon had a 13-fold higher risk of LBW delivery compared with noninfected pregnant women. In contrast, previous reports from Malawi [10, 33] and Ghana [31] showed no association between submicroscopic infections and LBW, since malaria parasitaemia of any density may have a harmful effect on fetal outcomes probably via maternal anaemia [34].

The present study has some limitations. The women were recruited at the time of delivery with no confirmed records of malaria infection during pregnancy. Unavailability of intermittent preventive treatment in pregnancy with sulphadoxine-pyrimethamine for the prevention of pregnancy associated malaria and submicroscopic malaria in pregnancy was another limitation. Furthermore, gestation was not evaluated using ultrasound examination to distinguish if LBW was due to infection only or as preterm birth.

## 5. Conclusion

The findings of this large sample size study clearly associated submicroscopic infections in any of the three compartments with an increased risk of maternal anaemia and LBW in women of all gravidities, and the findings show that the infections may have a harmful effect on pregnant women and their developing foetus.

Further research should investigate the detection of placental malaria from maternal peripheral blood during pregnancy because access to placental blood or tissue before delivery is practically impossible. Submicroscopic infections must be considered to ensure accurate estimation of the chances of successful malaria elimination.

## Figures and Tables

**Table 1:** Risk factors for submicroscopic parasitaemia in Sudanese women using univariate and logistic regression analyses.

Characteristics	*N* (%) of the total	Submicroscopic parasitaemia positive *N* (%)	Univariate analyses		Logistic regression analyses	
			OR (95% CI)	*P*	OR (95% CI)	*P*
*Site of collection*						
Ed-Dmazen Hospital	59 (13.2)	25 (17.0)	1.8 (1.1–3.2)	0.042	1.4 (0.8–2.3)	0.182
El-Roseures Hospital	103 (23.0)	40 (27.2)	1.5 (0.9–2.5)	0.060	1.5 (0.8–2.9)	0.149
Surgical complex	285 (63.8)	82 (55.8)	Reference		Reference	

*Age*, *years*^*∗*^	29.9 (4.5)	26.5 (4.7)	0.96 (0.9–4 1.0)	0.164	1.0 (0.9–1.1)	0.890

*Gravidity* ^*∗*^	3.4 (2.0)	2.3 (1.8)	0.95 (0.8–1.1)	0.326	0.9 (0.8–1.1)	0.646

*Residence*						
Rural	363 (81.2)	111 (75.5)	1.4 (1.1–1.8)	0.039	0.7 (0.4–1.3)	0.379
Urban	84 (18.8)	36 (24.5)	Reference		Reference	

*Education*						
<secondary level	313 (70.0)	126 (85.7)	3.6 (2.1–6.0)	<0.001	4.1 (2.3–7.4)	<0.001
≥secondary level	134 (30.0)	187 (19.8)	Reference		Reference	

*Antenatal care visits*						
Not attended	333 (74.5)	100 (68.0)	1.6 (1.1–2.5)	0.037	1.4 (0.9–2.7)	0.114
Attended	114 (25.5)	47 (32.0)	Reference		Reference	

*Use bednets*						
No	367 (82.1)	113 (76.9)	1.6 (1.1–2.7)	0.049	1.5 (0.9–2.7)	0.089
Yes	80 (17.9)	34 (23.1)	Reference		Reference	

*Sex of the newborn*						
Female	218 (48.8)	76 (51.7)	1.1 (0.8–1.7)	0.421	1.0 (0.7–1.6)	0.688
Male	229 (51.2)	71 (48.3)	Reference		Reference	

*N* = number; OR = odds ratio; CI = confidence interval. ^*∗*^mean (SD).

**Table 2:** Risk factors for anaemia in Sudanese women using univariate and logistic regression analyses.

Characteristics	*N* (%) of the total	Anaemia *N* (%)	Univariate analyses		Logistic regression analyses	
			OR (95% CI)	*P*	OR (95% CI)	*P*
*Site of collection*						
Ed-Dmazen Hospital	59 (13.2.)	15 (16.5)	1.9 (1.1–3.7)	0.011	1.5 (0.8–3.0)	0.192
El-Roseures Hospital	103 (23.0)	29 (31.9)	1.7 (0.8–3.3)	0.107	0.9 (0.3–2.1)	0.827
Surgical complex	285 (63.8)	47 (51.6)	Reference		Reference	

*Age*, *years*	29.9 (4.5)	27.1 (4.1)	1.0 (0.9–1.0)	0.713	0.9 (0.8–1.0)	0.485

*Gravidity*	3.4 (2.0)	3.7 (1.8)	1.0 (0.9–1.2)	0.113	1.2 (0.9–1.5)	0.077

*Residence*						
Rural	363 (81.2)	76 (83.5)	1.2 (0.6–2.2)	0.625	1.9 (0.9–4.0)	0.084
Urban	84 (18.8)	15 (16.5)	Reference		Reference	

*Education*						
<secondary level	313 (70.0)	78 (85.7)	3.0 (1.6–5.7)	<0.001	1.3 (0.6–3.4)	0.446
≥secondary level	134 (30.0)	13 (14.3)	Reference		Reference	

*Antenatal care visits*						
Not attended	333 (74.5)	60 (65.9)	1.6 (1.1–2.7)	0.043	1.3 (0.7–2.4)	0.380
Attended	114 (25.5)	31 (34.1)	Reference		Reference	

*Use bednets*						
No	367 (82.1)	69 (75.8)	1.6 (0.9–2.8)	0.092	1.0 (0.5–2.1)	0.880
Yes	80 (17.9)	22 (24.2)	Reference		Reference	

*Submicroscopic parasitaemia*						
Positive	147 (32.9)	75 (82.4)	18.4 (10.1–33.6)	<0.001	19.7 (10.3–37.8)	<0.001
Negative	300 (67.1)	16 (17.6)	Reference		Reference	

**Table 3:** Risk factors for low birth weight in Sudanese women using univariate and logistic regression analyses.

Characteristics	*N* (%) of the total	Low birth weight *N* (%)	Univariate analyses		Logistic regression analyses	
			OR (95% CI)	*P*	OR (95% CI)	*P*
*Site of collection*						
Ed-Dmazen Hospital	59 (13.2)	7 (11.9)	1.1 (0.5–2.5)	0.744	0.9 (0.3–2.1)	0.858
El-Roseures Hospital	103 (23.0)	9 (8.7)	1.6 (0.6–3.9)	0.301	0.9 (0.3–2.6)	0.971
Surgical complex	285 (63.8)	22 (7.7)	Reference		Reference	

*Age*, *years*	29.9 (4.5)	26.5 (3.3)	0.9 (0.9–1.0)	0.539	0.9 (0.8–1.0)	0.571

*Gravidity*	3.3 (1.9)	3.7 (1.8)	0.9 (0.8–1.1)	0.788	1.0 (0.7–1.3)	0.921

*Residence*						
Rural	363 (81.2)	31 (8.5)	1.0 (0.4–2.4)	0.951	1.1 (0.4–2.8)	0.805
Urban	84 (18.8)	7 (8.3)	Reference		Reference	

*Education*						
<secondary level	313 (70.0)	29 (9.3)	1.4 (0.6–3.0)	0.461	0.9 (0.3–2.4)	0.976
≥secondary level	134 (30.0)	9 (6.7)	Reference		Reference	

*Antenatal care visits*						
Not attended	333 (74.5)	19 (5.7)	3.3 (1.6–6.4)	0.001	2.8 (1.4–5.8)	0.004
Attended	114 (25.5)	19 (16.6)	Reference		Reference	

*Use of bednets*						
No	367 (82.1)	27 (7.4)	2.0 (0.9–4.2)	0.076	1.5 (0.6–3.3)	0.291
Yes	80 (17.9)	11 (13.8)	Reference		Reference	

*Sex of the newborn*						
Male	229 (51.2)	17 (7.4)	1.3 (0.6–2.5)	0.402	1.2 (0.5–2.4)	0.598
Female	218 (48.8)	21 (9.6)	Reference		Reference	

*Submicroscopic parasitaemia*						
Positive	147 (32.9)	22 (15.0)	3.1 (1.5–6.1)	0.001	2.7 (1.2–5.6)	0.008
Negative	300 (67.1)	16 (5.3)	Reference		Reference	

*N* = number; OR = odds ratio; CI = confidence interval.

## Data Availability

The data used to support the findings of this study are available from the corresponding author upon request.

## References

[1] World Health Organization (2016). *World Malaria Report 2016*.

[2] Brabin B. J., Romagosa C., Abdelgalil S. (2004). The sick placenta—the role of malaria. *Placenta*.

[3] Desai M., ter Kuile F. O., Nosten F. (2007). Epidemiology and burden of malaria in pregnancy. *The Lancet Infectious Diseases*.

[4] Stanisic D. I., Moore K. A., Baiwog F. (2015). Risk factors for malaria and adverse birth outcomes in a prospective cohort of pregnant women resident in a high malaria transmission area of Papua New Guinea. *Transactions of the Royal Society of Tropical Medicine and Hygiene*.

[5] Salanti A., Clausen T. M., Agerbæk M. Ø. (2015). Targeting human cancer by a glycosaminoglycan binding malaria protein. *Cancer Cell*.

[6] Sugiura N., Clausen T. M., Shioiri T., Gustavsson T., Watanabe H., Salanti A. (2016). Molecular dissection of placental malaria protein VAR2CSA interaction with a chemo-enzymatically synthesized chondroitin sulfate library. *Glycoconjugate Journal*.

[7] Mayor A., Moro L., Aguilar R. (2012). How hidden can malaria be in pregnant women? Diagnosis by microscopy, placental histology, polymerase chain reaction and detection of histidine-rich protein 2 in plasma. *Clinical Infectious Diseases*.

[8] Campos I. M., Maestre A., Franco-Gallego A., Uribe M. L., Cuesta C., Carmona-Fonseca J. (2011). Diagnosis of gestational, congenital, and placental malaria in Colombia: comparison of the efficacy of microscopy, nested polymerase chain reaction, and histopathology. *The American Journal of Tropical Medicine and Hygiene*.

[9] Singh N., Bharti P. K., Singh M. P. (2015). What is the burden of submicroscopic malaria in pregnancy in Central India?. *Pathogens and Global Health*.

[10] Rantala A.-M., Taylor S. M., Trottman P. A. (2010). Comparison of real-time PCR and microscopy for malaria parasite detection in Malawian pregnant women. *Malaria Journal*.

[11] Cohee L. M., Kalilani-Phiri L., Boudova S. (2014). Submicroscopic malaria infection during pregnancy and the impact of intermittent preventive treatment. *Malaria Journal*.

[12] Mohammed A. H., Salih M. M., Elhassan E. M. (2013). Submicroscopic *Plasmodium falciparum* malaria and low birth weight in an area of unstable malaria transmission in Central Sudan. *Malaria Journal*.

[13] Kapisi J., Kakuru A., Jagannathan P. (2017). Relationships between infection with *Plasmodium falciparum* during pregnancy, measures of placental malaria, and adverse birth outcomes. *Malaria Journal*.

[14] Arango E. M., Carmona-Fonseca J., Yanow S. K., Samuel R., Agudelo O. M., Maestre A. (2013). Molecular detection of malaria at delivery reveals a high frequency of submicroscopic infections and associated placental damage in pregnant women from Northwest Colombia. *The American Journal of Tropical Medicine and Hygiene*.

[15] Adegnika A. A., Issifou S., Verweij J. J. (2006). Microscopic and sub-microscopic *Plasmodium falciparum* infection, but not inflammation caused by infection, is associated with low birth weight. *The American Journal of Tropical Medicine and Hygiene*.

[16] Arango E., Maestre A., Carmona-Fonseca J. (2010). Effect of submicroscopic or polyclonal *Plasmodium falciparum* infection on mother and gestation product: systematic review. *Revista Brasileira de Epidemiologia*.

[17] Cottrell G., Moussiliou A., Luty A. J. F. (2015). Submicroscopic *Plasmodium falciparum* infections are associated with maternal anemia, premature births, and low birth weight. *Clinical Infectious Diseases*.

[18] Adam I., Khamis A. H., Elbashir M. I. (2005). Prevalence and risk factors for *Plasmodium falciparum* malaria in pregnant women of Eastern Sudan. *Malaria Journal*.

[19] Ali A. A., Elhassan E. M., Magzoub M. M., Elbashir M. I., Adam I. (2011). Hypoglycaemia and severe *Plasmodium falciparum* malaria among pregnant Sudanese women in an area characterized by unstable malaria transmission. *Parasites & Vectors*.

[20] Omer S. A., Idress H. E., Adam I. (2017). Placental malaria and its effect on pregnancy outcomes in Sudanese women from Blue Nile State. *Malaria Journal*.

[21] Adam I., A-Elbasit I. E., Salih I., Elbashir M. I. (2005). Submicroscopic *Plasmodium falciparum* infections during pregnancy, in an area of Sudan with a low intensity of malaria transmission. *Annals of Tropical Medicine & Parasitology*.

[22] Omer S., Khalil E., Ali H., Sharief A. (2011). Submicroscopic and multiple *Plasmodium falciparum* infections in pregnant Sudanese women. *North American Journal of Medical Sciences*.

[23] Plowe C. V., Djimde A., Bouare M., Doumbo O., Wellems T. E. (1995). Pyrimethamine and proguanil resistance-conferring mutations in *Plasmodium falciparum* dihydrofolate reductase: polymerase chain reaction methods for surveillance in Africa. *The American Journal of Tropical Medicine and Hygiene*.

[24] Snounou G., Viriyakosol S., Zhu X. P. (1993). High sensitivity of detection of human malaria parasites by the use of nested polymerase chain reaction. *Molecular and Biochemical Parasitology*.

[25] Fadlelseed O. E., Osman M. E., Shamseldin N. M., Elhussein A. B., Adam I. (2017). *Plasmodium falciparum* genotypes in matched peripheral, placental and umbilical cord blood in an area characterised by unstable malaria transmission in Eastern Sudan. *Heliyon*.

[26] Nwaefuna E. K., Afoakwah R., Orish V. N., Egyir-Yawson A., Boampong J. N. (2015). Effectiveness of intermittent preventive treatment in pregnancy with sulphadoxine-pyrimethamine against submicroscopic falciparum malaria in central region, Ghana. *Journal of Parasitology Research*.

[27] Okell L. C., Ghani A. C., Lyons E., Drakeley C. J. (2009). Submicroscopic infection in *Plasmodium falciparum*-endemic populations: a systematic review and meta-analysis. *The Journal of Infectious Diseases*.

[28] Okell L. C., Bousema T., Griffin J. T., Ouédraogo A. L., Ghani A. C., Drakeley C. J. (2012). Factors determining the occurrence of submicroscopic malaria infections and their relevance for control. *Nature Communications*.

[29] Mayengue P. I., Rieth H., Khattab A. (2004). Submicroscopic *Plasmodium falciparum* infections and multiplicity of infection in matched peripheral, placental and umbilical cord blood samples from Gabonese women. *Tropical Medicine and International Health*.

[30] Walker-Abbey A., Thuita L. H., Zhou A. (2005). Malaria in pregnant Cameroonian women: the effect of age and gravidity on submicroscopic and mixed-species infections and multiple parasite genotypes. *The American Journal of Tropical Medicine and Hygiene*.

[31] Mockenhaupt F. P., Bedu-Addo G., von Gaertner C. (2006). Detection and clinical manifestation of placental malaria in Southern Ghana. *Malaria Journal*.

[32] Saute F., Menendez C., Mayor A. (2002). Malaria in pregnancy in rural Mozambique: the role of parity, submicroscopic and multiple *Plasmodium falciparum* infections. *Tropical Medicine and International Health*.

[33] Mankhambo L., Kanjala M., Rudman S., Lema V. M., Rogerson S. J. (2002). Evaluation of the OptiMAL rapid antigen test and species-specific PCR to detect placental *Plasmodium falciparum* infection at delivery. *Journal of Clinical Microbiology*.

[34] Lone F. W., Qureshi R. N., Emanuel F. (2004). Maternal anaemia and its impact on perinatal outcome. *Tropical Medicine and International Health*.

